# Doxycycline Resistance and 16S rRNA Mutations in *Treponema pallidum*

**DOI:** 10.3201/eid3208.260433

**Published:** 2026-08

**Authors:** Mathew A. Beale, Michael Marks, Annie Luetkemeyer, Connie Celum, Matthew R. Golden, Lorenzo Giacani, Nicole A.P. Lieberman

**Affiliations:** Wellcome Sanger Institute, Hinxton, UK (M.A. Beale); London School of Hygiene and Tropical Medicine, London, UK (M. Marks); University of California San Francisco, San Francisco, California, USA (A. Luetkemeyer); University of Washington, Seattle, Washington, USA (C. Celum, M.R. Golden, L. Giacani, N.A.P. Lieberman)

**Keywords:** *Treponema pallidum*, syphilis, antimicrobial resistance, bacteria

**To the Editor:** We read with interest but also concern the article by Long et al. (*1*) describing putative doxycycline resistance–associated variants of the *Treponema pallidum* ribosomal RNA 16S gene. Determining if *T. pallidum* can become resistant to doxycycline is urgent, given its use for both prevention (i.e., doxy-PEP) and treatment of syphilis, especially amid ongoing shortages of benzathine penicillin G.

As the groups that generated most of the sequencing data reanalyzed in Long et al. (*1*), our analyses were unable to replicate the primary finding of a heterozygous G966T 16S mutation (*Escherichia coli* numbering) in 9 samples. Using standard pathogen genomics methods (Figure), we did not find the variant in any reanalyzed sample at an allele frequency exceeding background technical noise.

**Figure:**
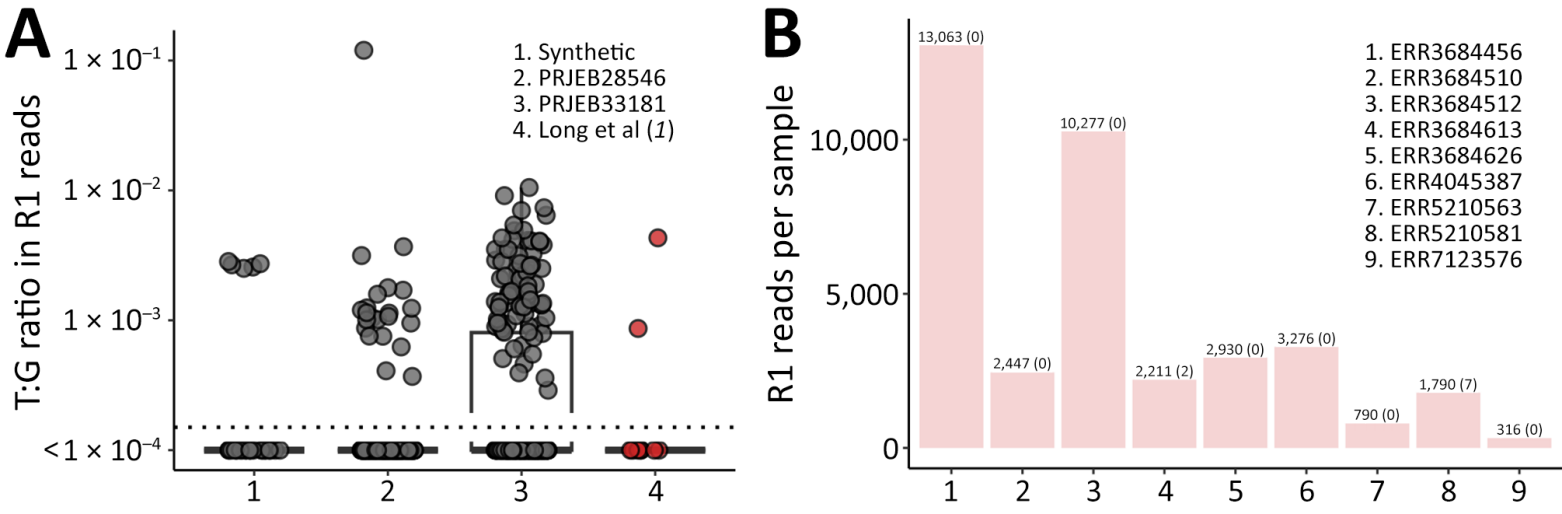
Ratio of G966T to wild-type G alleles among R1 reads of *Treponema pallidum*. We downloaded raw reads from the European Nucleotide Archive (BioProjects PRJEB28546 and PRJEB33181), which were re-assembled in Long et al. (*1*) and generated synthetic reads with the error profile of the Illumina HiSeq 2500from 29 high-quality *T. pallidum* assemblies from GenBank using Illumina ART (https://www.niehs.nih.gov/research/resources/software/biostatistics/art). We removed reads containing host sequences by using kraken 2 (https://github.com/DerrickWood/kraken2), performed quality and adaptor trimming with Trimmomatic version 0.39 (https://github.com/usadellab/trimmomatic), and filtered remaining read pairs to include only reads unambiguously arising from *T. pallidum* (taxid 160) using the standard kraken 2 16GB database with the default kmer length of 35. We isolated reads containing sequences from either of the rRNA loci by using bbduk (https://sourceforge.net/projects/bbmap) requiring a 21-mer match to the *T. pallidum* 16S locus sequence with 1 or 0 mismatches. For unambiguous identification and enumeration of the wild-type or G966T variant, R1 reads were grepped for perfect matches to the 51-base sequence centered on nt 966 (bold), equivalent to positions 232265 and 280700 in the SS14 reference sequence chromosome (CP004011): GGTGGAGCATGTGGTTTAATTCGAT**G**ATACGCGAGGAACCTTACCCGGGTT (wild-type) and GGTGGAGCATGTGGTTTAATTCGAT**T**ATACGCGAGGAACCTTACCCGGGTT (G966T), and the reverse complement of each. A) T:G ratio at rRNA position 966 in samples with >100 R1 reads (n = 622). Each point represents a sample; points below the dotted line contain 0 G966T reads. Boxplots represent medians and interquartile ranges. Samples identified in Long et al. do not have a G966T allele frequency exceeding the technical noise from PCR and sequencing. B) Total R1 reads containing wild-type and mutant sequence in 9 samples identified by Long et al. The total reads are shown for each sample and the number of reads supporting the G966T mutation is shown in parentheses.

Of note, >98% of publicly available *T. pallidum* genomes were generated from clinical specimens by metagenomic sequencing using hybrid capture probes. That method retains conserved non–*T. pallidum* DNA, such as ribosomal operons from other bacteria, in the sample (*2*,*3*), and can introduce errors when merging fragmented DNA reads into consensus sequences. Such artifacts may be incorporated into consensus *T. pallidum* genomes available from public resources such as pubMLST (https://pubmlst.org) or GenBank and misinterpreted as real mutations. Although Long et al. (*1*) reported filtering for reads arising from *Treponema*, we and others have shown the necessity of more stringent methods, such as competitive mapping versus related ribosomal sequences (*2*) or requiring near-identity to known *T. pallidum* rRNA sequences (*4*), to avoid inadvertent reporting errors.

The development of doxycycline resistance by *T. pallidum* could have devastating consequences for the control of syphilis and is being closely monitored by clinicians and scientists. We are encouraged by increasing genomic surveillance of *T. pallidum*, providing a mechanism for early detection of the emergence of resistance-associated variants.
